# A model free method of predicting transient dynamics in anaerobic digestion

**DOI:** 10.1098/rsif.2024.0059

**Published:** 2024-03-27

**Authors:** Christopher M. Heggerud, Alan Hastings

**Affiliations:** Department of Environmental Science and Policy, University of California Davis, Davis, CA, USA

**Keywords:** anaerobic digestion, transient dynamics, empirical dynamical modelling, dynamical systems, forecasting

## Abstract

Transient dynamics pose unique challenges when dealing with predictions and management of ecological systems yet little headway has been made on understanding when an ecological system might be in a transient state. As a start we consider a specific model, here focusing on a canonical model for anaerobic digestion. Through a series of simplifications, we analyse the potential of the model for transient dynamics, and the driving mechanisms. Using a stochastic analogue of this model, we create synthetic ecological data. Thus, combining our understanding of the deterministic transient dynamics with the use of empirical dynamical modelling, we propose several new metrics to indicate when the synthetic time series is leaving a transient state.

## 1. Introduction

Throughout the historical modelling literature an emphasis has been placed on asymptotic, or terminal dynamics. However, a significant amount of recent literature suggests that important information is overlooked by only studying the stationary dynamics. For these reasons, a growing focus has been placed on the study of transient dynamics. In this sense, transient dynamics are any, primarily nonlinear, non-asymptotic dynamic that occurs on ecologically relevant timescales. Several authors have introduced novel ideas and concepts towards the study of transient dynamics including classification of types of long transients [1,2] and the mathematical classification of transient points [3]. Much of these classifications arises from the study of dynamical systems which have generally helped gain deeper understanding of ecological systems from a mathematical perspective [4,5]. Fortunately, the mathematical underpinnings are readily transferred to ecological systems, and the theory greatly aids in other study areas, such as stochastic systems [6] and empirical studies [7,8].

In this paper, our goal is to use the current knowledge of transient dynamics, from a deterministic stand point, to form predictions about transient behaviour in ecological time series. A potentially useful tool to accomplish this goal is the empirical dynamical modelling (EDM) framework, and in particular the sequential locally weighted global linear map (S-map). The S-map was popularized by Sugihara and colleagues to forecast variables from a given ecological time series [9]. EDM aims to transform time-series data into some reconstruction of a dynamical attractor that describes the behaviour of a particular dynamical system. Much of the theory involved in EDM is mathematical in nature and stems from Takens' work [10] in which Takens proposed that the behaviour of the underlying dynamical system can be uncovered through a single variable with delayed coordinates. That is, the behaviour of an entire dynamical system can be recreated through the observations of a single variable. Moreover, if multi-dimensional time series are observed then the reconstruction becomes even more convincing, or potentially requires less data [11–13].

Since the majority of the underlying theory involved in EDM is mathematical, most of the methods can be modified for various applications. For example, Brias & Munch [14] have used EDM to propose management strategies in multi-species systems, Tsonis *et al.* [15] have used the S-map to test if a system is highly nonlinear or stochastic and Wasserman *et al.* [16] have used the S-map to determine the temporally changing interaction strengths among competing fish species. Additionally, the EDM literature has led to many interesting theoretical advances in ecological forecasting [17], dealing with missing data or non-uniform sampling times [18] and stability of potential fixed points [11,19]. One particular application that begs for further study is the use of EDM to give early warning signals of regime shifts or changes in the dynamics as popularized by Scheffer *et al.* [20,21]. For example, Rypdal & Sugihara [22] use EDM to predict the magnitude of dengue fever outbreaks in San Juan, Puerto Rico by predicting eigenvalues of sequentially computed Jacobians from EDM. However, many of these results predict a qualitative change in the dynamics, such as a bifurcation point or a change in stability. Making similar predictions for transient dynamics, that change due to the inherent nature of system, i.e. no changes in parameters, is an important next step towards understanding and managing ecological systems. In this paper, we apply the EDM framework to predict a transition between transient dynamics and steady-state dynamics. This work is novel as it provides predictions of transient dynamic end times, which is yet to be done, and provides a new application of the EDM framework. Furthermore, this work will help to advance the transient, theoretical ecology and EDM literature.

In our particular study, we use anaerobic digestion (AD) as an example of an ecological system that is high dimensional, where data collection of many dimensions is feasible, and that also commonly exhibits transient dynamics. These aspects make AD a useful system for our study, since the EDM framework can benefit from high dimensionality. AD is the process by which bacteria break down simple substrates into useful products in the absence of oxygen. Applications of AD are diverse and expanding but typically include sewage/wastewater treatment and biogas production [23–25]. Although the dynamics of AD are quite complex [23], several studies have been put forward to simplify the system in an attempt to yield tractable mathematical models. This mathematical effort has had an important impact in understanding AD and has led to advances in engineering, control and promising mitigation aid for the current climate emergency [26–28]. Although these papers have been pivotal in understanding the dynamics of AD, they neglect the study of any transient behaviour. In both the laboratory and wastewater processes, transient dynamics are often observed [29,30] and can be important for understanding when certain controls should or should not be implemented to maintain the function of the AD systems. In this work, we offer a novel mechanistic explanation of the transient dynamics exhibited in AD systems.

**Figure 1.**
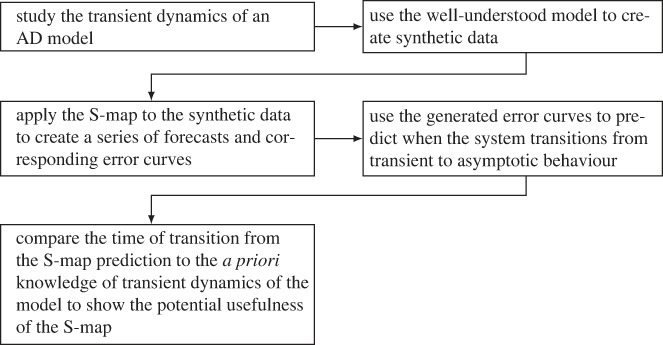
Flowchart describing the overall process of formulating our method.

In §2, we discuss the process of AD in more detail and propose a system of ordinary differential equations to model it. We further make several simplifying assumptions with justification. The resulting reduced model is then non-dimensionalized, leading to uncovering a separation of timescales. Analysis on both the fast and slow timescales is performed in §3 to fully understand the potential dynamics and mechanisms driving these dynamics. This deeper understanding of the dynamics, and in particular the understanding of the transient dynamics, becomes critical in §5 where we attempt to predict these transient dynamics. In §4, a stochastic analogue of the simplified AD model is given and used to create synthetic data to be used in the analysis of our method. Such an approach can be beneficial for establishing a method using ecological time-series data as we are able to confer any results back to the underlying deterministic system [6,17]. In §5, we introduce the S-map, an EDM method, and use it to forecast variables from the generated synthetic data. These forecasts then lead to the production of error curves corresponding to the prediction error. Using the underlying dynamical systems theory involved in both the S-map and transient dynamics we use these error curves to create several metrics that predict when a transient dynamic is ending. The use of such error curves also establishes a novel approach in understanding the underlying dynamical properties of a time series. We then compare our predictions with our knowledge of the transient dynamics of the deterministic system to compare and assess these methods. A general summary of the paper is given in figure 1.

## 2. A model for anaerobic digestion

To start, we introduce a mechanistic model for the AD of simple substrates into useful biogas. The model will then be simplified and analysed to gain in-depth understanding of the transient dynamics of the biological system.

Although the entire AD process is very complex, a series of simplifications lead to a tractable system of differential equations that holds similar qualitative dynamics, mainly those that are transient in nature. Based on previous simplifications [27,28] we consider five categorical variables to describe AD. We assume that *S*_1_ represents compounds of simple substrates such as sugars, *S*_2_ represents the volatile fatty acids, and *S*_3_ the concentration of pH reducing ammonia. Lastly, we track bacterial concentrations that correspond to the bacteria that break down simple substrates (*X*_1_) into volatile fatty acids (VFAs) and the ones that break down VFAs into biogas (*X*_2_), respectively. *X*_1_ and *X*_2_ may be referred to as acidogenic and methanogenic bacteria, respectively. Our model is then derived from the following reaction equation:

2.1 $$\begin{array}{rl} & y_{1}S_{1}\overset{r_{1}}{\to }X_{1}+y_{2}S_{2}+y_{4}S_{3}+y_{5}CO_{2}\\ \;\mathrm{and}\; & y_{3}S_{2}\overset{r_{2}}{\to }X_{2}+y_{6}CO_{2}+y_{7}CH_{4}, \end{array}\}$$

where *r*_1_ = *μ*_1_(*S*_1_)*X*_1_ and *r*_2_ = *μ*_2_(*S*_2_, *S*_3_)*X*_2_ are the reaction rates defined by the bacteria growth rates and *y*_*i*_ are the yield constants described in table 1.

**Table 1.** Parameters and their definitions for reaction (2.1) and resulting differential equation (2.2).

parameter	definition	units
*D*	chemostat gilution rate	d^−1^
*S*^0^	concentration of *S*_1_ input	g l^−1^
*y*_1_	yield constant (degradation)	(*g*[*S*_1_]/*g*[*X*_1_])
*y*_2_	yield constant (production)	(mmol[*S*_2_]/*g*[*X*_1_])
*y*_3_	yield constant (consumption)	(mmol[*S*_2_]/*g*[*X*_2_])
*y*_4_	yield constant (production)	(mmol[*S*_3_]/*g*[*X*_2_])
*k*_1_	decay rate	d^−1^
*k*_2_	decay rate	d^−1^
*H*_1_	h.s.c for *S*_1_ degradation	g l^−1^
*H*_2_	h.s.c for *S*_2_ consumption	mmol l^−1^
*μ*_1,max_	max acidogenic biomass growth rate	d^−1^
*μ*_2,max_	max methanogenic biomass growth rate	d^−1^

h.s.c stands for half saturation constant.

Inhibition dependent on the level of *S*_3_ is considered in several papers [27,28,31]. However, here we assume that the inhibition of all reactions by ammonia (*S*_3_) is negligible. This argument can be supported in certain cases of AD and is deemed reasonable for our study [32]. Thus, although ammonia (*S*_3_) is still being produced, it has no effect on the systems dynamics and is ignored for our study. Furthermore, we assume that additional factors that may be inhibiting methanogenesis are negligible. Additionally, by considering the reaction (2.1) in a chemostat setting, where the simple substrate, *S*_1_, is continuously being added we arrive at the following model:

2.2 $$\begin{array}{rl} & {\dot{S}}_{1}=D(S^{0}-S_{1})-y_{1}\frac{\mu_{1,\max}S_{1}}{H_{1}+S_{1}}X_{1},\\ & {\dot{S}}_{2}=-DS_{2}+y_{2}\frac{\mu_{1,\max}S_{1}}{H_{1}+S_{1}}X_{1}-y_{3}\frac{\mu_{2,\max}S_{2}}{H_{2}+S_{2}}X_{2},\\ & {\dot{X}}_{1}=-DX_{1}-k_{1}X_{1}+\frac{\mu_{1,\max}S_{1}}{H_{1}+S_{1}}X_{1}\\ \;\mathrm{and}\; & {\dot{X}}_{2}=-DX_{2}-k_{2}X_{2}+\frac{\mu_{2,\max}S_{2}}{H_{2}+S_{2}}X_{2}, \end{array}\}$$

with growth rates

2.3 $$\mu_{1}(S_{1})=\frac{\mu_{1,\max}S_{1}}{H_{1}+S_{1}}$$

and

2.4 $$\mu_{2}(S_{2})=\frac{\mu_{2,\max}S_{2}}{H_{2}+S_{2}}.$$

**Figure 2.**
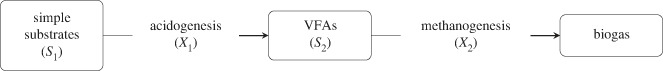
Schematic of the simplified AD process describing model (2.2).

This system now has four state variables and is hence slightly more simplified. Furthermore, a schematic is shown in figure 2 describing the simplified AD process. The parameters and their definitions are given in table 1. Certain qualitative aspects of the dynamics are undoubtedly lost when inhibition is neglected; however, transient dynamics still occur and are qualitatively similar, indicating that inhibition is not the important factor for studying transient dynamics, which is the main focus of this paper.

### 2.1. Non-dimensional model

Next, we perform a non-dimensionalization of system (2.2). The non-dimensionalization allows for a clearer understanding of which parameters are of particular interest and may be regarded as perturbation parameters. Additionally, the non-dimensionalization reduces the number of model parameters and rescales all state variables to be closer in magnitude while maintaining all qualitative properties of the original model. The non-dimensionalization of (2.2) is given in appendix A and ultimately leads to the following non-dimensional model:

2.5 $$\begin{array}{rl} & \dot{u}=\alpha -\epsilon u-\beta \frac{u}{1+u}x,\\ & \dot{v}=-\epsilon v+\frac{u}{1+u}x-\frac{v}{1+v}y,\\ & \dot{x}=-\epsilon x-\sigma_{1}x+\frac{u}{1+u}x\\ \;\mathrm{and}\; & \dot{y}=-\epsilon (1+\sigma_{2})y+\epsilon \omega \frac{v}{1+v}y. \end{array}\}$$

**Figure 3.**
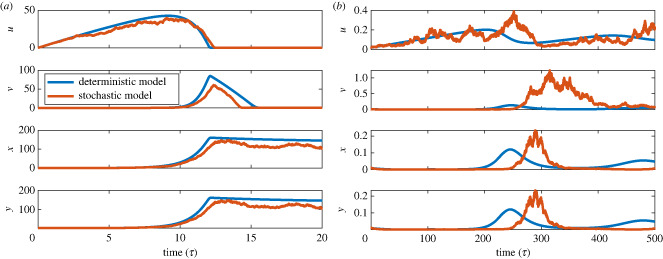
Two sample simulations of model (2.5) (blue) and its stochastic extension outlined in §4.1 (orange). Parameter values are given in table 2 with *α* = 6 (*a*) and *α* = 0.001 (*b*).

A summary of the dimensionless parameters is given in table 2 and sample dynamics of model (2.5) are given in figure 3 in blue for two sets of parameter values. A few assumptions are made about the dimensional parameters which results in a further simplified dimensionless model. First, we assume that the chemostat is slowly being diluted, i.e. the dilution rate, *D*, is very small but that the input concentration, *S*^0^ is high. These are easily controllable parameters in most settings, readily justifying these assumptions. We further assume that the generational timescale of the acidogens, *X*_1_ (*x*) is small, thus both the growth rate, *μ*_1_, and the decay rate, *k*_1_, are relatively large but similar in magnitude. We further assume that the methanogens, *X*_2_ (*y*), have a small decay rate, *k*_2_. It is typical that the acidogenic process is faster moving than the methanogenic process and thus we argue that these assumptions are reasonable. These assumptions lead us to the following conclusions about the non-dimensional parameters and system. First, since *D* is small and *μ*_1_ is large, the non-dimensional parameter $\epsilon =D/\mu_{1}$ is small and is considered a perturbation parameter that, as we will show, leads to a separation of timescales. Secondly, since *S*^0^ is large, we argue that the parameter $\alpha =\epsilon S^{0}/H_{1}$ is of an intermediate order. Finally, given our assumptions about *k*_1_ and *k*_2_, the parameters *σ*_1_ = *k*_1_/*μ*_1_ and *σ*_2_ = *k*_2_/*D* are also of comparable order. For the remainder of this paper the parameter values for model (2.5) are set to the values given in table 2 unless otherwise noted. The values are chosen to be biologically reasonable, but also so that transient dynamics are exhibited.

**Table 2.** Dimensionless variables and parameters for system (2.5).

parameter	definition	value
ϵ	*D*/*μ*_1_	≪1
*α*	*S*^0^*D*/*H*_1_*μ*_1_	0.001–6
*β*	*y*_1_/*cH*_1_	0.336
*σ*_1_	*k*_1_/*μ*_1_	0.1
*σ*_2_	*k*_2_/*D*	0.0667
*ω*	*μ*_2_/*D*	4.27

## 3. Fast–slow dynamics of the anaerobic digestion model

We now discuss in detail how the small parameter ϵ leads us to a separation of timescales and eventually allows for the understanding of the transient dynamics. First, system (2.5) is represented as the classical fast–slow system as follows:

3.1 $$\dot{x}=f\;(x,y;\epsilon )$$

and

3.2 $$\dot{y}=\epsilon g(x,y;\epsilon ),$$

where *x* is a vector representing the fast variables, and *y* the slow. By using the geometric singular perturbation theory we assume the fast dynamics of *x* occur on the timescale *τ* and the slow dynamics of *y* occur on the timescale s=ϵτ. These assumptions allow us to approximate the dynamics of (2.5) by assuming that the system can be broken down into two simpler subsystems. Furthermore, the dynamics of *x* on the slow timescale are restricted to a manifold defined by *f*(*x*, *y*; 0) = 0 while *y* is treated as a constant on the fast timescale. Fortunately, the theory provided by Fenichel allows us assert that the dynamics of the approximation is a reasonable representation of the full system [33,34].

### 3.1. Slow dynamics

For the slow dynamics, we first perform a change of variables given by s=ϵτ. Here, *s* represents slow time, where *τ* is the fast timescale. Letting ϵ→0 gives the first governing differential-algebraic system of the dynamics of the slow timescales given by

3.3 $$\begin{array}{rl} & 0=\alpha -\beta \frac{u}{1+u}x,\\ & 0=\frac{u}{1+u}x-\frac{v}{1+v}y,\\ & 0=-\sigma_{1}x+\frac{u}{1+u}x\\ \;\mathrm{and}\; & y^{\prime }=-(1+\sigma_{2})y+\omega \frac{v}{1+v}y, \end{array}\}$$

where the ′ refers to the derivative with respect to *s*. Note that the solution to the algebraic component is dependent on *y*. Thus, dynamically the solution for *u*, *v* and *x* on the slow timescale will change as the value of *y* changes as to satisfy the algebraic constraint. Eventually, a steady state in all variables is achieved.

### 3.2. Fast dynamics

The fast dynamics occur on the timescale given by *τ*. Again, by letting ϵ→0, we arrive at the following reduced system of equations describing the fast dynamics:

3.4 $$\begin{array}{rl} & \dot{u}=\alpha -\beta \frac{u}{1+u}x,\\ & \dot{v}=\frac{u}{1+u}x-\frac{v}{1+v}y,\\ & \dot{x}=-\sigma_{1}x+\frac{u}{1+u}x\\ \;\mathrm{and}\; & \dot{y}=0. \end{array}\}$$

In the first-order approximation of the fast system, *y* acts as a constant. Thus, we can analyse this subsystem using the classical methods. It is easy to show that only one equilibrium,

3.5 $$(u^{*},v^{*},x^{*})=\left(\frac{\sigma_{1}}{1-\sigma_{1}},\frac{\alpha }{\beta y-\alpha },\frac{\alpha }{\beta \sigma_{1}}\right),$$

exists for system (3.4) and that the eigenvalues describe its long-term dynamics. The eigenvalues of the linearized system around (u*, v*, x*) are given in equation (B 1). Furthermore, it is shown in appendix B that these eigenvalues have negative real parts for our chosen parameter ranges and imply that (u*, v*, x*) is a stable equilibrium of the fast subsystem.

### 3.3. Transient dynamics of the fast system

Although the fast–slow analysis in the above two sections is useful in understanding the overall dynamics of the system, many aspects of the transient dynamics are still uncovered. That is, up to this point we only understand what the critical manifold looks like and how the dynamics change on the critical manifold. With respect to transient dynamics, the way the critical manifold is approached is often interesting and helps to better explain the mechanisms driving transient dynamics. In this light, we further analyse the fast subsystem motivating the results shown in later sections.

We begin with the fast subsystem (3.4), and assume that all of our initial conditions are relatively small, except perhaps *y*. This assumption allows us to, for only a short amount of time, claim that many of the terms in (3.4) are negligible, or in the very least, do not contribute to the qualitative transient dynamics that initially occur. This further implies that for a small amount of initial time the transient dynamics are mainly governed by the following:

3.6 $$\begin{array}{cc} & \dot{u}=\alpha ,\\ & \dot{v}=0,\\ & \dot{x}=0\\ \;\mathrm{and}\; & \dot{y}=0. \end{array}\}$$

Assuming *α* is relatively large we expect to see an abrupt increase in *u*, initially. Note that the transient dynamics are approximated by (3.6) until *u* becomes large enough that the terms (*u*/(1 + *u*))*x* are no longer negligible. Conveniently, this time is computed from the solution to the simple equation, i.e. for small initial conditions, *u*(*τ*) = *u*_0_ + *ατ*. Now, since the initial growth of *u* is fast we define a time, *τ*_1_, such that for *τ* > *τ*_1_, *u*/(1 + *u*) ≈ 1 and for *τ* < *τ*_1_ the dynamics are approximated by system (3.6). Furthermore, *τ*_1_ can be computed as *τ*_1_ = (*u*_*tol*_ − *u*_0_)/*α*, where *u*_*tol*_ is the value of *u* such that *u*/(1 + *u*) ≈ 1 within a given error tolerance.

For large *u* the system is now able to respond by producing acidogens at a significant rate and in turn producing VFAs. In other words, for *τ* immediately larger than *τ*_1_, *x* and *v* are still small but increasing. Thus, our system can be thought to be governed by

3.7 $$\begin{array}{rl} & \dot{u}=\alpha -\beta x,\\ & \dot{v}=x-\frac{v}{1+v}y,\\ & \dot{x}=(1-\sigma_{1})x\\ \;\mathrm{and}\; & \dot{y}=0 \end{array}\}$$

for *τ*_1_ < *τ* < *τ*_2_, where *τ*_2_ is the point that *u* stops increasing and the assumption that *u*/(1 + *u*) ≈ 1 is no longer reasonable.

Again, the time *τ*_2_ is computed explicitly as the point in time that *u* is no longer increasing, i.e. *α* − *βx* = 0. Thus, by solving the decoupled approximate system we compute *τ*_2_ = ln(*α*/*βx*_0_) + *τ*_1_. Finally, for *τ* > *τ*_2_ all of our previous approximations become unreasonable and we assume that the dynamics are governed by the original fast subsystem (3.4). Since we understand the dynamics for the *τ* < *τ*_2_ it is easy to deduce that for time *τ* > *τ*_2_ the dynamics will monotonically approach equilibrium values.

**Figure 4.**
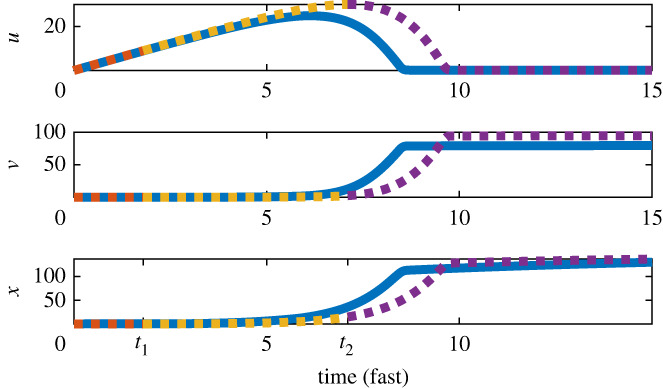
The comparison of the approximation of the transient dynamics described in §3.3 with the numerical simulation is shown here. The approximation is given with the dashed curves and the critical times are given on the time axis.

In summary, for 0 < *τ* < *τ*_1_, the dynamics are approximated by (3.6), for *τ*_1_ ≤ *τ* ≤ *τ*_2_ the dynamics are approximated by (3.7), and for *τ* > *τ*_2_ the dynamics are monotonically approaching equilibrium governed by (3.4). Figure 4 shows the comparison of the above approximation of our dynamics with the full system, in which we show that the approximation is qualitatively sufficient for understanding the transient dynamics.

## 4. Synthetic data

In this section, we create a stochastic analogue of system (2.2) to produce a number of synthetic time series. We then treat these time series as synthetic ecological data which will be used to give our main result in §5. Additionally, using the knowledge of the deterministic system (2.5) gained in §3.3, we numerically compute the time in which the transient dynamic ends for each time series. Furthermore, the dynamics of the non-dimensional model (2.5) are equivalent to the dynamics of the original model (2.5) through a scaling of the state variables discussed in appendix A.

### 4.1. Creation of synthetic data

Here, we create the stochastic analogue of system (2.2) to produce synthetic data for later analysis. In doing so we assume that each growth or death/conversion process has some inherent noise associated with it. We do not consider additional noise in the data sampling, although this is no doubt a great concern for ecological applications.

We generate our synthetic data following the ideas of Reimer *et al.* [6]. First, we assume that there is a core process for each state variable described by the following system:

4.1 $$\begin{array}{rl} & \frac{d\mu }{dt}=D(S^{0}-S_{1})-y_{1}\frac{\mu_{1,\max}S_{1}}{H_{1}+S_{1}}X_{1},\\ & \frac{d\nu }{dt}=-DS_{2}+y_{2}\frac{\mu_{1,\max}S_{1}}{H_{1}+S_{1}}X_{1}-y_{3}\frac{\mu_{2,\max}S_{2}}{H_{2}+S_{2}}X_{2},\\ & \frac{d\nu }{dt}=-DX_{1}-k_{1}X_{1}+\frac{\mu_{1,\max}S_{1}}{H_{1}+S_{1}}X_{1}\\ \;\mathrm{and}\; & \frac{d\chi }{dt}=-DX_{2}-k_{2}X_{2}+\frac{\mu_{2,\max}S_{2}}{H_{2}+S_{2}}X_{2}. \end{array}\}$$

We then assume that the true change of our state variables is the summation of the change described by the core process plus a unique multiplicative stochastic process. Thus, the true change of the state variable is given by

4.2 $$\begin{array}{rl} & dS_{1}=d\mu +dW_{S_{1}},\\ & dS_{2}=d\nu +dW_{S_{1}},\\ & dX_{1}=d\chi +dW_{X_{1}}\\ \;\mathrm{and}\; & dX_{2}=d\psi +dW_{X_{2}}, \end{array}\}$$

where the following probability density function (PDF), defined similarly for all variables, describes the stochastic process and ensures numerical positivity and a non-zero probability of extinction in finite time:

4.3 $$p(dW_{x}(t)=w|x(t))=\left\{ \begin{array}{cc} N(w|0,\sigma^{2}x{\left(t\right)}^{2}dt)\; & x(t)+w>0,\;\\ \Phi \left(-\frac{1}{\sigma \sqrt{dt}}\right)\; & x(t)+w=0,\;\\ 0\; & x(t)+w<0.\; \end{array}\right.$$

Furthermore, *N*(*w*|*μ*, *σ*^2^) is the PDF of the normal distribution and Φ((x−μ)/σ) is its cumulative distribution function. Note that the variances of the PDF of the stochastic noise terms in (4.2) are dependent on state variables and are thus equivalent to multiplicative noise. Moreover, this conditional probability density function ensures that when the state variables are large, the stochastic process represents the typical Brownian motion with multiplicative noise. However, when the state variables become small there is non-zero probability that the population becomes zero and collapses with a zero probability that it becomes negative.

**Figure 5.**
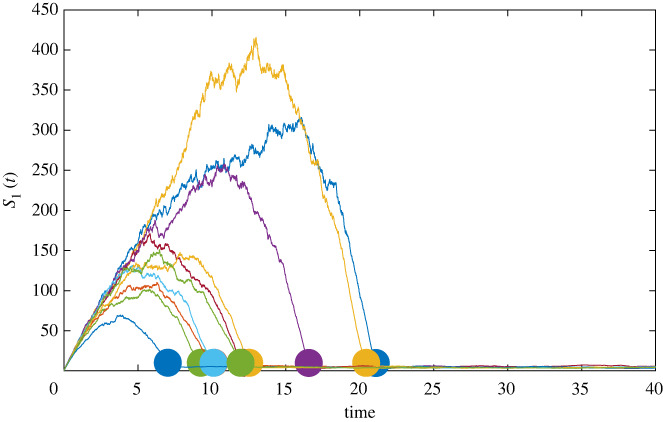
Samples of synthetic data given by system (4.2). The large dots are labelled at time *t*_*k*,end_ for each realization to represent the computed time in which transients end. These time series were generated using *σ* = 0.65, Δ*t* = 0.01 with all other parameters as in table 3.

We produce several realizations of the stochastic dynamics governed by system (4.2) for uniformly distributed random initial conditions with the following restrictions: *S*_1_(0) ∈ (0, 1), *S*_2_(0) ∈ (0, 1), *X*_1_(0) ∈ (0, 0.5) and *X*_2_(0) ∈ (0, 1). These stochastic simulations are then treated as our synthetic ecological data for future analysis and several time-series data are plotted in figure 5 as an example for the state variable *S*_1_. Furthermore, the stochastic analogue is presented in conjunction with the dynamics of the deterministic model in figure 3. The parameter values chosen for the simulations are given in appendix C.1. The goal of this work is to develop a method to detect the transition from transient dynamic to the exponential decay towards the equilibrium. Although the exponential decay could still be considered a transient dynamic, we argue that it is of less interest, as this type of dynamic is dominated by linear terms and exhibits no nonlinear behaviour. Initial conditions that are close to the equilibrium approach the equilibrium in a simple exponential decay (i.e. a linear dynamic). We intentionally chose initial conditions that are sufficiently far from the equilibrium point so that a nonlinear transient dynamic occurs. Additionally, as discussed in §3, small initial conditions are assumed for our deterministic study of transient dynamics of the model.

**Table 3.** Parameters, their definitions and values used to generate synthetic data in §4.

parameter	definition	value
*D*	chemostat dilution rate	0.055
*S*^0^	concentration of *S*_1_ input	700
*y*_1_	yield constant (degradation)	42.14
*y*_2_	yield constant (production)	116.5
*y*_3_	yield constant (consumption)	268
*k*_1_	decay rate	0.1
*k*_2_	decay rate	0.001
*H*_1_	h.s.c for *S*_1_ degradation	10
*H*_2_	h.s.c for *S*_2_ consumption	9.28
*μ*_1,max_	max acidogenic biomass growth rate	0.5
*μ*_2,max_	max methanogenic biomass growth rate	0.0064
*σ*	noise level of stochastic simulation	0.0005–5
*h*	number of historical data points to train S-map forecast	2–20
Δ*t*	time step of a given synthetic time series	0.05-1
*δ*_*a*_, *δ*_*r*_	error tolerance thresholds	10^−3^–10^2^

h.s.c stands for half saturation constant.

### 4.2. Transients in the synthetic data

Based on our *a priori* study of the transients of the deterministic model (2.5), we can determine at which time the transient phase of our synthetic time series ends. To this end, we assume that once the dynamics are near the equilibrium value computed from the deterministic model that our transients end. As a simplification, we assume that the transients of interest are observed in the state variable *S*_1_. Thus, we assume the transients end when *S*_1_ begins to approach the equilibrium point. That is, when the synthetic data, given by the sequence *S*_1_(*t*_1_), …, *S*_1_(*t*_*i*_), …, *S*_1_(*t*_*n*_), where *t*_*i*_ is an ordered sequence, shows the distance between *S*_1_ and $S_{1}^{*}$ is small we claim the transient has ended. Thus, the time the transient dynamics end, denoted as *t*_*k*,end_, is given by the smallest value of *t*_*i*_ that satisfies $|S_{1}(t_{i})-S_{1}^{*}|<\delta_{eq}$. Note that for the parameter values used (see table 3 in appendix C.1) $S_{1}^{*}=4.49$. For noisier time series, we allow *δ*_*eq*_ to be larger. The exact values we chose for *δ*_*eq*_ are described in appendix C.2. Now, for each synthetic time series generated we can compute, with reasonably high confidence, the time (*t*_*k*,end_) the transient dynamics end. In figure 5, we show a sample of the transient dynamics with random initial conditions as described above. The large dots represent the point in time in which our measure indicates the transient dynamics end. Our overall goal is to predict these points using only the synthetic data assuming no prior knowledge of the dynamics.

## 5. Predicting the end of a transient dynamic

In this section, we introduce the EDM tool, the S-map, and use its properties to predict the end of a transient dynamic in our synthetic time-series data. We discuss this new method and apply to two different scenarios of time-series data; a control time series, and historic time-series data.

### 5.1. S-map introduction

Here, we use a method of forecasting called sequentially computed Jacobian coefficients, or the S-map for short. The S-map forecasts an ecological variable from a given historical or control time series. The S-map uses training data to describe a dynamical attractor which is then used to form a prediction from a point in state space. Furthermore, data points from the time series are weighted accordingly to their proximity from the point of prediction. In particular, the S-map is used to forecast a variable *p* time units from time t*. That is, it obtains a prediction for the value of the target variable *Y*(t* + *p*) from using some training time series with *k* data points. The time series may contain multiple observations and is given the notation {*D*(*t*_*i*_)} where *D*(*t*_*i*_) is an *E* dimensional vector consisting of system variables, or a vector containing our data for time *t*_*i*_. The vector *D* may also contain embedded data if higher dimensionality is desired. For example, the data vector at time *t*_*i*_ may be written as *D*(*t*_*i*_) = (*d*_1_(*t*_*i*_), *d*_2_(*t*_*i*_), …, *d*_*E*_(*t*_*i*_)). In our particular case *D*(*t*_*i*_) = (*S*_1_(*t*_*i*_), *S*_2_(*t*_*i*_), *X*_1_(*t*_*i*_), *X*_2_(*t*_*i*_)). We assume that the time-series data are given at regular intervals with no missing points such that *t*_*i*+1_ = *t*_*i*_ + *p* for all *i*. In ecological monitoring, this is rarely the case; however, one can use one of several imputation methods to produce an approximate and regular time series, and this is not the focus of this paper. Furthermore, the target variable *Y* is the forecasted value of a variable contained in *D*. Without loss of generality, we assume that we are interested in predicting the variable $d_{*}(t)$ which is an element of the vector *D* (*Y* is the forecast of $d_{*}$). The forecast is given by first assigning weights to each of the *k* training data points based on its distance to the target point in the attractor manifold and is completely irrespective of time. That is,

5.1 $$w_{i}=\exp\left(\frac{-\theta \|D(t_{i})-D(t^{*})\|}{\bar{d}}\right),$$

where *θ* = 5 is the measure of nonlinearity, $\bar{d}$ is the average distance from point *D*(t*) given as

5.2 $$\bar{d}=\frac{1}{n}\sum_{\;j=i}^{k}\|D(t_{j})-D(t^{*})\|,$$

and ‖ · ‖ is the Euclidean norm. Now the forecast mapping is given by

5.3 $$Y(t^{*}+p)=\sum_{\;j=1}^{E}C_{j}\cdot d_{j}(t^{*}),$$

where the vector *C* = (*C*_1_, *C*_2_, …, *C*_*E*_) is the solution to the linear equation

5.4 B=A⋅C.

The matrices *A* and *B* represent the weighted state space vectors and the weighted future value of the target variable, respectively. In general, we define

5.5 $$A_{ij}=w_{i}d_{j}(t_{i})\;\text{and}\;B_{i}=w_{i}d_{*}(t_{i+1}),$$

where $d_{*}$ is the target variable.

Although the S-map is typically used for forecasting it can be used in many fashions. In this paper, we show that based on the error of the forecasts we can gain insight as to the dynamics of our system. In particular, we argue that a forecast with higher error implies that the time series is not near its attractor, whereas a forecast with lower error implies that the forecasting is being made on, or near the attractor. In this light, one can say that a high forecasting error indicates a transient state, or transition from a transient state, based on our knowledge of the transient dynamics obtained in the previous section and general dynamical systems theory.

### 5.2. Prediction of transients from synthetic data

We now use the S-map to forecast the dynamics based on the synthetic data. We consider two practical situations in which data would be gathered. The first case is that of a single time series in which only historical data is available, similar to monitoring data. The second case is when an entire control time series exists, as common in the laboratory setting or where an entire experiment/observation has been done previously. The key difference is that in the first case, we use a number of historical data points from the same time series to train the S-map forecast, whereas in the second case we use a single control time series in its entirety to train the S-map forecast of a different time series. We then use the S-map to create prediction error curves; absolute error or absolute relative error. The absolute error curve is computed as the absolute difference between the S-map prediction and the actual value of the time series at time *t*_*i*_ given as

5.6 $$R_{\mathrm{abs}}(t_{i})=|Y(t_{i})-d_{*}(t_{i})|.$$

The absolute relative error is computed as the absolute error relative to the values of the time series

5.7 $$R_{\mathrm{rel}}(t_{i})=\left|\frac{Y(t_{i})-d_{*}(t_{i})}{d_{*}(t_{i})}\right|.$$

**Figure 6.**
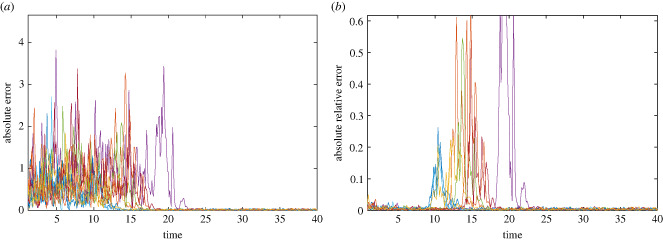
Errors of prediction as a function of time. (*a*) A plot of the smoothed absolute error as a function of time for 10 sample time series. The point in which the error stays below a threshold corresponds to the end of the transient. (*b*) A plot of the smoothed absolute relative error as a function of time for 10 sample time series. The point in which the error is above some threshold and is also a local maximum corresponds to the end of the transient dynamic. Both use the parameter values *h* = 20, Δ*t* = 0.1 and *σ* = 0.1.

Since absolute relative error can be sensitive for small predicted values and absolute error can be sensitive for larger predicted values, we consider both error measures for completeness although generally absolute relative error is deemed to be a better measure. We further argue that the transient dynamic ends if either the absolute error becomes lower than some predetermined threshold *δ*_*a*_, or when the absolute relative error becomes greater than some predetermined threshold *δ*_*r*_. Due to the S-map's decreased prediction error near an equilibrium (figure 6*a*), we argue that the first point in time the absolute error becomes less than *δ*_*a*_ indicates the beginning of the equilibrium phase, and thus the end of the transient. On the other hand, when using absolute relative error, the S-maps prediction error will ‘spike’ when the dynamics transition from the transient phase to the equilibrium phase (figure 6*b*). Hence, the first point in time the absolute relative error is greater than *δ*_*r*_ corresponds to the end of the transient dynamics. The choice of *δ*_*a*_ and *δ*_*r*_ is dependent on the level of noise in the system, the values of the state variables in equilibrium, and the degree of nonlinearity in the dynamics. Thus, in this study we do not focus on how to choose these thresholds, but rather show that there is range of thresholds in which the end of the transient can be reasonably predicted.

We furthermore consider two types of synthetic time series data which we use for our predictions: monitoring data and control data. In the case of monitoring data, we assume that we have a single synthetic time series in which we do not know the outcome of the dynamics. We use previous data points of the monitored synthetic time series to train the S-map for forecasting. In the case of control data, we assume that we have one entirely realized synthetic time series that is used to train the S-map. The S-map then uses the control time series to forecast from a single observation of an entirely separate synthetic time series, or experiment.

Finally, to compare the values of the predicted transient end time with the transient end time computed from the knowledge of the underlying deterministic system, as in §4.2, we use the coefficient of determination and Pearson’s correlation coefficient. In particular, we compute the coefficient of determination (*R*^2^) with respect to the line *y* = *x* and not the standard line of best fit. Thus, in this case, a value of *R*^2^ = 1 suggests that the S-map perfectly predicts the computed transient end time, whereas decreasing values indicate poorer predictions. The Pearson correlation coefficient gives a measure of how well correlated our predicted transient end times and the computed times are. In this sense, a Pearson correlation coefficient equal to one suggests that the S-map prediction and the computed transient end time of each time series are perfectly correlated, but does not necessarily suggest the prediction is good. That is, high correlation could mean our prediction consistently under-predicts, or over-predicts the computed transient end time. However, these results are still useful in evaluating our methods.

#### 5.2.1. Monitoring data

We first consider the scenario where we are dealing with a single time series, similar to monitoring data. For the given time series, we assume that the data points are equally spaced in time (i.e. *t*_*i*_ − *t*_*i*−1_ = Δ*t*, for all *i*), and that the previous *h* time points are used to train the S-map in order to make the forecast. For example, we wish to forecast from the current point, *t*_*j*_. To obtain *Y*(*t*_*j*_ + Δ*t*), we use the data vectors *D*(*t*_*j*−*h*_), …, *D*(*t*_*j*−1_) as the training data for the S-map forecast. Here, *Y*(*t*_*j*_ + Δ*t*) is the forecast of *S*_1_ and each data vector contains the information *D*(*t*_*i*_) = (*S*_1_(*t*_*i*_), *S*_2_(*t*_*i*_), *X*_1_(*t*_*i*_), *X*_2_(*t*_*i*_)). If *j* < *h* then we use every historical point in the time series. We forecast every reasonable time point in a similar fashion, thus ending up with forecasts based only on historical time points for each time *t*_2_, …*t*_*k*_. For each forecast, we compute the absolute error and the absolute relative error as given in equations (5.6) and (5.7), respectively. In this sense, we have created new time series, representing the respective errors, that will be used to make conclusions about the transient dynamics. To fully use these created error curves, we smooth out some of the fluctuations by computing the Gaussian weighted moving average over a fixed number of previous values. The results of the smoothing are plotted in figure 6 for 10 sample time series.

We consider 10 different noise levels (*σ* as in (4.3)) and for each noise level we generate 1000 time series. To show the utility of this method, we do not always use the entire time series to make forecasts. That is, to explore how sparseness of data influence our methods outcome we consider different values for the time step between data points used (Δ*t*), and the maximum number of historical data points used to train the forecast (*h*). For example, we extract a subset of data points from the original time series to create our synthetic data where *t*_*i*_ − *t*_*i*−1_ = Δ*t*. Furthermore, to make the forecast from time *t*_*j*_ to time *t*_*j*_ + Δ*t* the S-map is trained on the previous *h* data points in the synthetic time series, i.e *t*_*j*−*h*_ · · · *t*_*j*−1_ (if *j* ≤ *h* we use all previous time points). For each synthetic time series, we generate smoothed error curves that represent the absolute error and the absolute relative error between the forecast and the true value of the synthetic time series. We then use the generated error curves, as in figure 6, to predict the point in time the transients end, denoted ${\tilde{t}}_{k,\mathrm{end}}$.

We argue that when the absolute error, as plotted in figure 6*a*, is less than a certain predetermined tolerance, *δ*_*a*_, the dynamics are not transient and are sufficiently near some attractor. The prediction is made for several values of *δ*_*a*_ for each time series.

Moreover, we make similar predictions using the absolute relative error as shown in figure 6*b*. However, when using the absolute relative error curve to predict the end of the transient phase, merely predetermining a threshold is not sufficient because the S-map yields smaller values of absolute relative error while in the transient phase, and only increases when the dynamics leave the transient phase. This is due to the idea that the transient dynamic can be viewed as weak, or temporary, attraction to some region in state space that is eventually overpowered by another attractor [1]. While the dynamics are near this transient psuedo-attractor the absolute relative error is low. Thus, when using absolute relative error the S-map can highlight this transient psuedo-attractor, resulting in a lower prediction error. The absolute error cannot capture this transient psuedo-attractor as well, due its limited complexity and sensitivity to larger numbers. However, as the dynamics leave the transient phase, the prediction error should increase until the dynamics become near an attractor again. This transition from one attractor to another corresponds to a spike in the absolute relative error as seen in figure 6*b*. Using this knowledge, we can predict the end of the transient phase. We claim that the transient ends once the absolute relative error become larger than some threshold value, *δ*_*r*_, and is a local maximum of the error curve.

**Figure 7.**
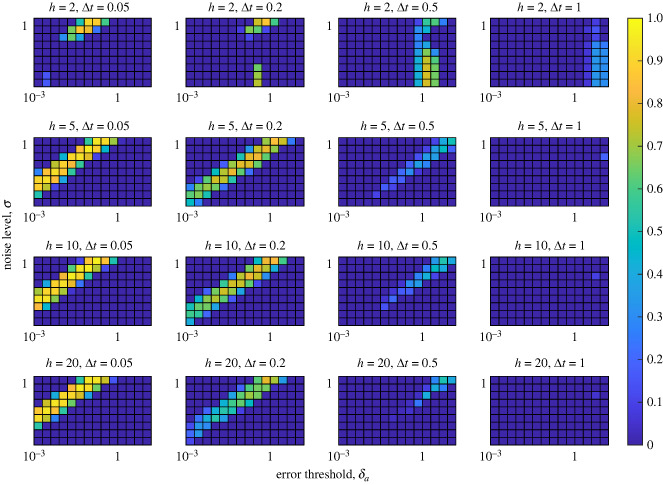
Prediction accuracy comparing the computed transient end with the S-map-forecasted transient end time based on the absolute error curves. The heat map represents the value of *R*^2^ relative to the line *y* = *x*. Each panel corresponds to a fixed number of historical points used to train the forecast (*h*) and a fixed time step between data points (Δ*t*). The *y*-axis of each panel represents the noise level of the simulated synthetic time series (*σ*), and the *x*-axis represents the error tolerance threshold (*δ*_*a*_).

**Figure 8.**
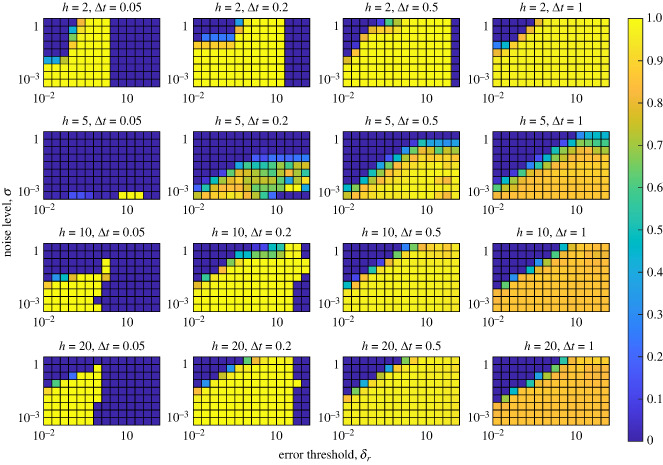
Prediction accuracy comparing the computed transient end with the S-map-forecasted transient end time based on the absolute relative error. The heat map represents the value of *R*^2^ relative to the line *y* = *x*. Each panel corresponds to a fixed number of historical points used to train the forecast (*h*) and a fixed time step between data points (Δ*t*). The *y*-axis of each panel represents the noise level of the simulated synthetic time series (*σ*), and the *x*-axis represents the error tolerance threshold (*δ*_*r*_).

The accuracy of the prediction for 1000 synthetic time series over 10 different noise levels (*σ*), 15 different error tolerances (*δ*_*a*_ and *δ*_*r*_), four different step lengths (Δ*t*) and four different numbers of historical data points used for training (*h*) are given in figures 7, 8, 13 and 14. Figures 7 and 8 use the coefficient of determination (*R*^2^) as the measure of prediction accuracy using the absolute and absolute relative errors, respectively. Figures 13 and 14 use Pearson’s correlation coefficient as a measure of prediction accuracy using the absolute and absolute relative errors, respectively. Additional details regarding the proportion of successfully made predictions, (i.e. instances where the error tolerances are not passed result in failed predictions) and sample scatter plots of predicted vs. computed transient end times are given in appendix D.

Using this approach, we note that in some cases the absolute relative error will not exceed *δ*_*r*_, or the absolute error will not be less than *δ*_*a*_, thus our methods will not yield a prediction. However, as shown in figures 17 and 18, we see that there are large regions in both the noise level and error tolerances for which predictions are made successfully.

#### 5.2.2. Control data

To understand the potential applicability of our method we now assume that a single ‘control’ time series exists in its entirety to act as the S-map training dataset. By contrast, in the previous section, we used only historical data from a single time series to make the prediction. Here, we use an entire time series to make the prediction given the current state of a completely different time series, but with assumed identical model parameters.

**Figure 9.**
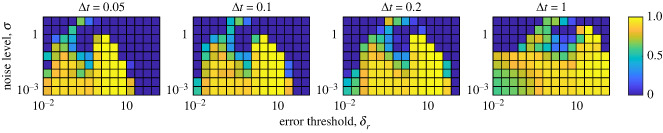
Prediction accuracy comparing the computed transient end with the S-map-forecasted transient end time based on the absolute relative error. The heat map represents the value of *R*^2^ relative to the line *y* = *x*. Each panel corresponds to a fixed time step (Δ*t*) used in the control data to train the forecast. The *y*-axis of each panel represents the noise level of the simulated synthetic time series (*σ*), and the *x*-axis represents the error tolerance threshold (*δ*_*r*_).

We compute the S-map prediction as follows. First, we establish our control synthetic time series denoted by $D_{c}(t_{1}),\;\ldots ,\;D_{c}(t_{n}),$ where $D_{c}(t_{i})=(S_{1}^{c}(t_{i}),S_{2}^{c}(t_{i}),X_{1}^{c}(t_{i}),X_{2}^{c}(t_{i}))$ for all *i*. This control time series is the same for all forecasts at each noise level. Again, once we establish the variable we are predicting we proceed with the calculation of *Y*(*t*_*j*_ + Δ*t*) for all *t*_*j*_ ∈ {*t*_1_ · · · *t*_*n*−1_}. For each *t*_*j*_ the S-map is trained using the control time series, *D*_*c*_, and we assume that a vector of the current state of our system is given as *D*(*t*_*j*_) = (*S*_1_(*t*_*j*_), *S*_2_(*t*_*j*_), *X*_1_(*t*_*j*_), *X*_2_(*t*_*j*_)). Here, *Y*(*t*_*j*_ + Δ*t*) is the forecast of *S*_1_. To this end, every forecast is made using the same training dataset, unlike in the previous section. As before, we generate 1000 time series for each noise level *σ*, with random initial conditions as described in §4.1. Again, we take a subset of each time series to create the synthetic time series such that the synthetic data points are spaced by Δ*t* time units. We then predict *Y*(*t*_*j*_ + Δ*t*) for all *j* in the sequence and for each synthetic time series using the S-map trained on an entire single control time series. Each prediction *Y*(t* + *p*) is compared against the true value from the synthetic time series. From this, we compute the relative absolute error curve described in equation (5.7). The error curves here are similar in nature to what is shown in figure 6 and are not shown. As before, we use the same threshold *δ*_*r*_ and predict that the transient dynamic ends when the absolute relative error is larger than *δ*_*r*_ for the first time and represents a local maximum of the absolute relative error curve. To measure of the accuracy of our predictions, we give the coefficient of determination, *R*^2^, relative to the line *y* = *x* in figure 9. Here, *R*^2^ is a function of noise level (*σ*) and error threshold *δ*_*r*_. Also, in figure 9, we provide this measure four different time steps (Δ*t*) in the data to represent varying sparseness of the data. Furthermore, to complement the results in figure 9 we show, in appendix D, a similar heat map for Pearson’s correlation coefficient (figure 19), a sample scatter plot comparing predicted with computed transient end times (figure 20) and heat map showing the proportion of synthetic time series where a prediction was successfully made (figure 21).

## 6. Discussion

Recent studies have emphasized the importance of transient dynamics for ecology. Much of the scientific literature on transient dynamics revolves around interesting mathematical arguments. In particular, the classification of types of transient dynamics [1,2], the study of tipping points and regime shifts, and early warning signals all build on concepts from dynamical systems. In parallel, many efforts have focused on predicting a change in dynamics due to bifurcations from parameter changes or perturbations, with critical slowing down theory playing a prominent role [21,35–38]. In more detail, critical slowing down is the phenomenon that as a system approaches a bifurcation point, or a tipping point, it recovers from perturbations slower. For example, these perturbations could be the natural perturbations of an ecological system and, due to the slow response of the system, critical slowing down would then be signalled by an increased dependence of the future state on the previous state (i.e. higher auto-correlation) [21]. Moreover, even deep learning methods have been established to predict tipping points and provide details regarding the dynamics beyond the transitions [39]. However, each of these methods revolves around the assumption that the critical transition occurs due to tipping points, or something akin to a bifurcation. Using such methods to predict transitions due to reasons other than bifurcations or critical transitions can lead to incorrect results since the prediction relies on statistical patterns than can also emerge from nonlinear dynamics, such as abrupt large fluctuations [40]. Furthermore, few studies have focused on predicting transient dynamics when the underlying deterministic process and parameters are not changing in time. In this paper, we offer a novel study of interpreting and predicting dynamical changes caused by transients, rather than bifurcations, using ecological data and the concepts involved with EDM. An important area of future work would be to establish comparable methods that can predict these transitions spurred on by either tipping points or transients.

**Figure 10.**
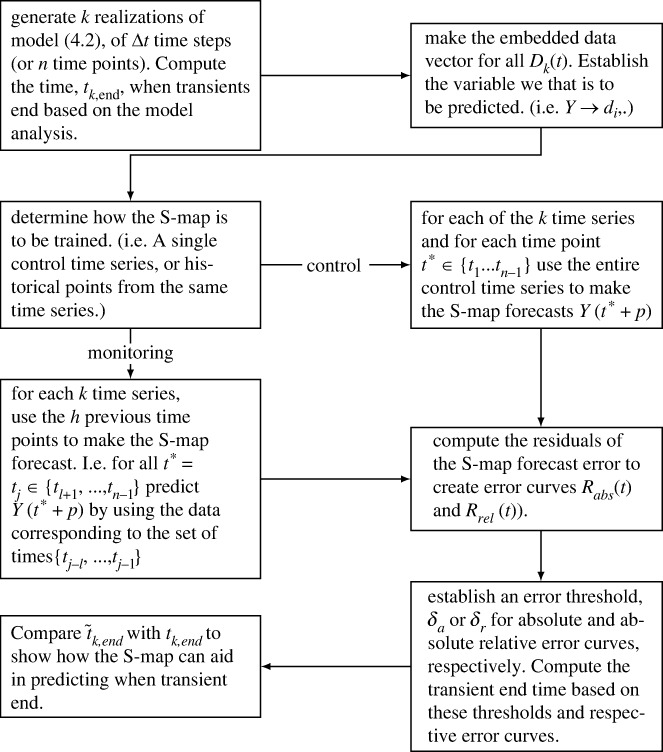
General workflow of how we use synthetic data to predict when a time-series leaves the transient state.

**Figure 13.**
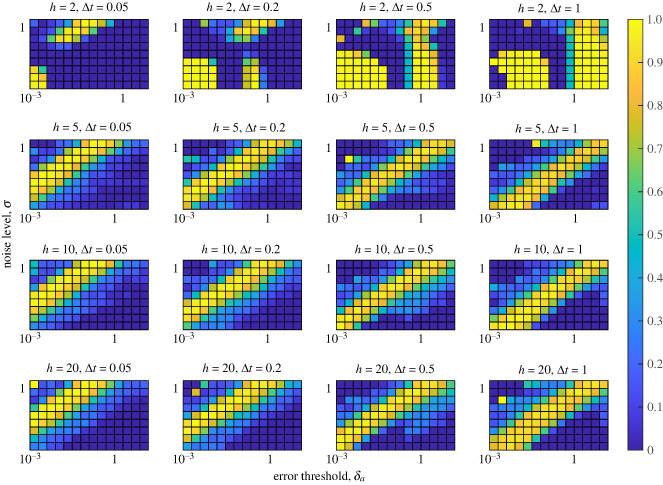
Prediction accuracy comparing the computed transient end with the S-map-forecasted transient end time based on the absolute error curves. The heat map represents the value of Pearson’s correlation coefficient. Each panel corresponds to a fixed number of historical points used to train the forecast (*h*) and a fixed time step between data points (Δ*t*). The *y*-axis of each panel represents the noise level of the simulated synthetic time series (*σ*), and the *x*-axis represents the error tolerance threshold (*δ*_*a*_).

**Figure 14.**
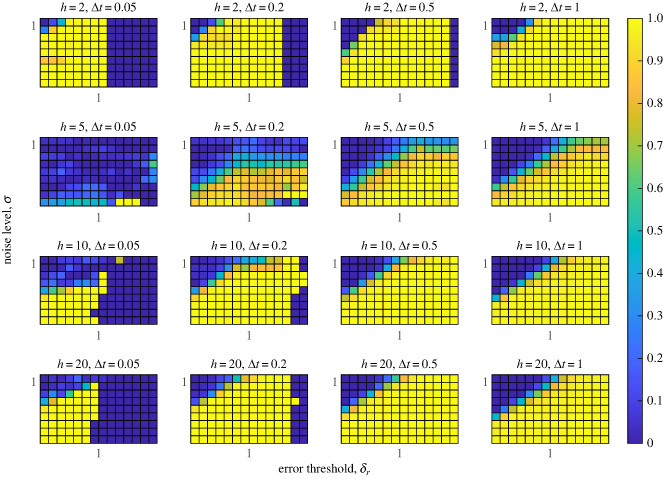
Prediction accuracy comparing the computed transient end with the S-map-forecasted transient end time based on the absolute relative error curves. The heat map represents the value of Pearson’s correlation coefficient. Each panel corresponds to a fixed number of historical points used to train the forecast (*h*) and a fixed time step between data points (Δ*t*). The *y*-axis of each panel represents the noise level of the simulated synthetic time series (*σ*), and the *x*-axis represents the error tolerance threshold (*δ*_*r*_).

Due to the complexity of the problem, we begin with a specific ecological system rather than starting with a general approach. The specific system we focused on, AD, is an important process for wastewater treatment and biogas production, and shows promising advances for dealing with the current climate emergency. Additionally, AD is biologically well studied, the focus of many mathematical modelling studies, a high-dimensional system and is well known to exhibit transient behaviour as shown in our model (2.5). For these reasons, we deemed AD a good specific system to begin with. However, AD is a highly complex process and the original formulation of our model, the ADM1 model [23] would consist of 32 state variables making modelling the entire process a difficult task. Through a series of simplifications and idealizations the complex ADM1 model is reduced to the model studied here [27,28]. Moreover, simplifications such as non-dimensionalization and fast–slow analysis enabled us to uncover the main drivers of the transient dynamics of this particular model as shown in figure 4. In general, the analytic study of transient dynamics in AD systems is important to understand the driving mechanisms, and we have offered novel insights in that regard. These insights lead to the main results of this paper presented in §5. Given the knowledge of the system gained in §3, we were then able to develop tools to predict when the transient phase of the dynamics will end. Using knowledge of the transient dynamics, we formulated several metrics based on the prediction errors of the EDM tool, the S-map. We argued that prediction error is relatively low when the dynamics are on, or near an attractor or region in state space that is regularly observed. Thus, when the prediction error is high, we concluded that the dynamics are not near an attractor and assume the dynamics are in a transient state. A work flow of this method is presented in figure 10. This relation of such error curves to the underlying dynamical properties of time series is a novel application of EDM and could lead to further insights in future studies.

**Figure 15.**
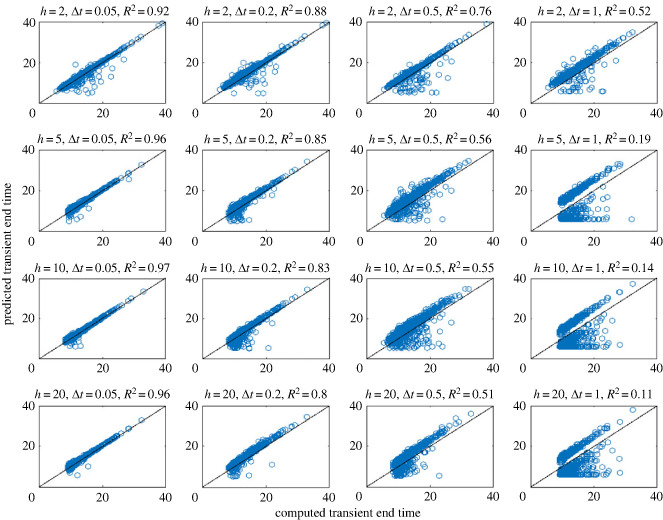
A sample scatter plot from each panel in figure 7 with the maximum value of *R*^2^ such that our method yielded a prediction for more than half of the synthetic time series.

**Figure 16.**
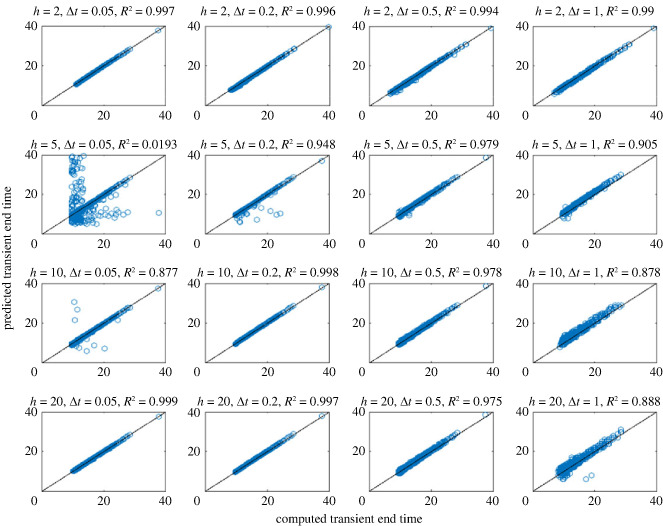
A sample scatter plot from each panel in figure 8 with the maximum value of *R*^2^ such that our method yielded a prediction for more than half of the synthetic time series.

**Figure 17.**
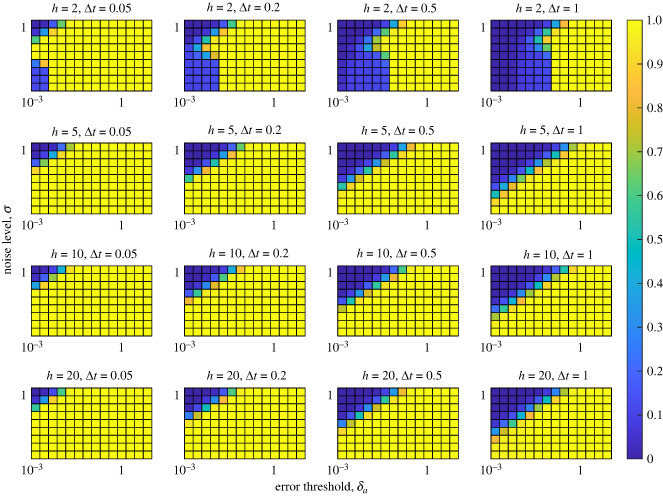
The proportion of generated synthetic time series in which our S-map method, using absolute error, successfully made a prediction of the end of the transient dynamics is shown by the heat map. This figure complements figures 7 and 13.

**Figure 18.**
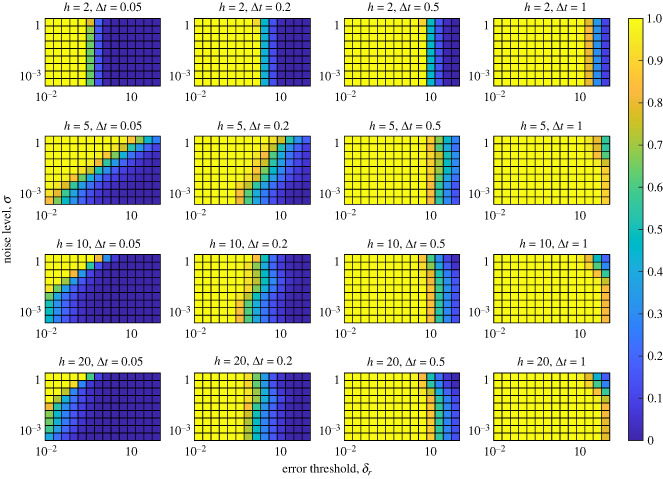
The proportion of generated synthetic time series in which our S-map method, using absolute relative error, successfully made a prediction of the end of the transient dynamics is shown by the heat map. This figure complements figures 8 and 14.

**Figure 19.**
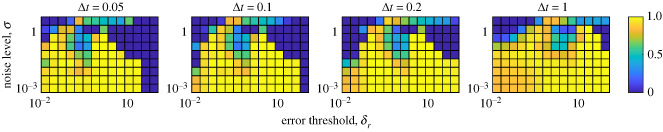
Prediction accuracy comparing the computed transient end with the S-map-forecasted transient end time based on the absolute relative error curves. The heat map represents the value of Pearson’s correlation coefficient. Each panel corresponds to a fixed time step (Δ*t*) used in the control data to train the forecast. The *y*-axis of each panel represents the noise level of the simulated synthetic time series (*σ*), and the *x*-axis represents the error tolerance threshold (*δ*_*r*_).

**Figure 20.**
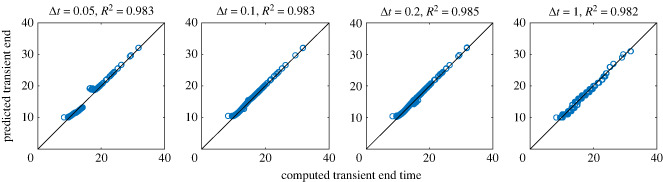
A sample scatter plot from each panel in figure 9 with the maximum value of *R*^2^ such that our method yielded a prediction for more than half of the synthetic time series.

**Figure 21.**
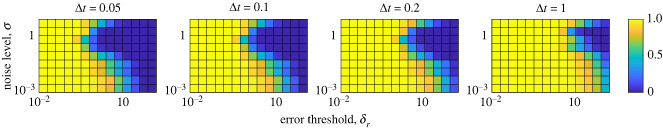
The proportion of generated synthetic time series in which our S-map method, using absolute relative error, successfully made a prediction of the end of the transient dynamics is shown by the heat map. This figure complements figures 9 and 19.

Our results suggest that the prediction of transient end times is possible using the EDM framework. In figure 7, we show that for smaller step sizes, and intermediate numbers of data points used to train the S-map, good predictions can be made. However, we note that for larger step sizes the accuracy is decreased, regardless of the number of data points trained on. This is not a surprising result, as larger time steps often correspond to larger prediction errors, or noise. However, increasing the number of data points used to train the method is noted to not increase the performance of the method, especially for larger step sizes. This is probably due to the over-representation of trajectories that do not inform a shift in dynamics. That is, more points are used to train the model that do not correspond to significant changes in the dynamics biasing the prediction against transitions. This keeps the error high past the point the steady state is reached or decreases the error while the dynamics are on the transient attractor. This claim is also supported by the correlation plots shown in figures 15 and 16 with the consistent scattered underestimation or slight overestimation of transient end times. Figures 8 and 9 suggest that the absolute relative error is more useful at predicting transient end times as it provides larger ranges of method parameters that yield high *R*^2^ values. However, in certain cases, this metric falls short in that the absolute relative error may not exceed the prescribed error threshold *δ*_*r*_ resulting in a failed prediction. Although this is a limitation, figures 18 and 21 show a reasonable range for which most predictions are made successfully. Additionally, certain trends in these figures bring useful insight. The low *R*^2^ values in figure 8 for *h* = 5, Δ = 0.05 suggest that intermediate numbers of historical data points can result in poorer predictions due to the over-representation of local data points. Also, the slight bimodality with respect to *δ*_*r*_ observed in figure 9 suggest that intermediate error thresholds decrease the prediction accuracy. This is probably explained by the inherent nonlinearities in the error curves which lead to spikes that do not represent the spike at the end of the transient. These spikes can exceed the error threshold for intermediate thresholds, even though there may be a larger spike at a later time corresponding to the true transient end time.

In this work, we argue that the end of the transient dynamic is signalled by certain error thresholds, *δ*_*a*_ or *δ*_*r*_. In particular, we argue that the transient dynamics end when the absolute error is low, signalling the dynamics are near the equilibrium, or when the absolute relative error spikes, suggesting the dynamics are quickly approaching the equilibrium. As it stands, these definitions are well suited for establishing a method of predicting the end of transient dynamics but how to select specific values of each threshold has not been linked to any biological reasoning and are only argued from a mathematical perspective. This is an unfortunate gap in this work; however, our results do suggest that accurate predictions can be made for a relatively large range of thresholds suggesting that future applications of this method are certainly possible. Moreover, the way we define the end of transient was based on our knowledge of the deterministic dynamics and could be defined in other ways, such as when the dynamics begin to return to (or stop moving away from) the equilibrium point, or when the first difference of the dynamics get close to zero. Regardless of how we define the end of the transient we conjecture that the accuracy of our predictions will not change due to the flexibility of the S-map and the general relationships between prediction error and proximity to attractors. Another aspect not studied in this work is the proximity of initial conditions to the equilibrium. Here, we chose only random initial conditions such that a nonlinear transient dynamic is exhibited (i.e. not just a decay towards equilibrium). For initial conditions near the equilibrium point our methods could still predict when the dynamics become within a certain range of the equilibrium or suggest that no transient dynamic occurs. This is not a limitation of the method, as dynamics that start near an equilibrium could be argued to not exhibit any transient dynamic at all. Hence, to focus on predicting the end of nonlinear transient dynamics we restrict our initial conditions to be sufficiently small and far away from the equilibrium point.

In future studies, the use of embedded coordinates [10,11] and a rigorous study of the dimensionality of such systems would be useful to improve efficiency in data collection of transient systems and to uncover insights regarding transient dynamics in existing time-series data that have limited dimension and quality. In particular, this work could be extended to the study of the entire complex ADM1 model [23] in order to predict the transient behaviour of a single monitored variable. The requirements of this extension are minimal, seeking only monitoring data for those variables of interests. However, the confidence of obtained results would depend on the frequency in which those variables are obtained as described here by the parameter Δ*t*.

In general, the importance of understanding transient dynamics from ecological data is manifested in the timing of management decisions [41]. In particular, manipulation of an ecological system can often alter the transient dynamics, but understanding these alterations poses certain additional challenges. For example, increased noise can either induce or destroy long transient dynamics [6] and perturbations can cause larger than anticipated population changes [42]. Thus, we offer methods to help confirm whether or not a system is in its transient state before ecological intervention is implemented in hopes to limit undesirable outcomes.

This work continues to build the tools and frameworks required to deeply understand transient phenomena in ecology. In particular, the overall concept of this work is to develop tools analogous to early warning signs for bifurcations [35]. That is, based on ecological time series and mathematical intuition we have proposed a rigorous, repeatable method of predicting the end of a transient phase. Although the end of the transient could be visually or computationally recognized by observing the time series in its entirety, the work presented here gives a reproducible and consistent method to ascertain the end of a transient dynamic. Furthermore, this method does not require the observation of a time series in its entirety, rather, only historical data or a full control time series is required. However, the method is limited to predicting when a known transient dynamic will end via an approach to a stable attractor or the repulsion from a pseudo attractor (i.e. ghost attractor or saddle node), and is not capable of predicting whether or not a transient dynamic will occur in general.

Even though we focused on AD for many keys reasons and presented these methods and concepts in a relatively simple specific ecological system with synthetic data, this work can be extended to understand transient dynamics in ecological systems where either the ecological data contains more noise or has a less obvious transient dynamic. Additionally, systems known to exhibit unrepeated transient behaviour, such as algal blooms [4], coral reefs [43], fish abundances [44] and food-web interactions [45], could have been the focus of this study and could furthermore be ecological systems where our methods are applied. Thus, this work is an important stepping stone towards understanding transients in ecological data, monitoring and more complex systems.

## Figures and Tables

**Figure 11.**
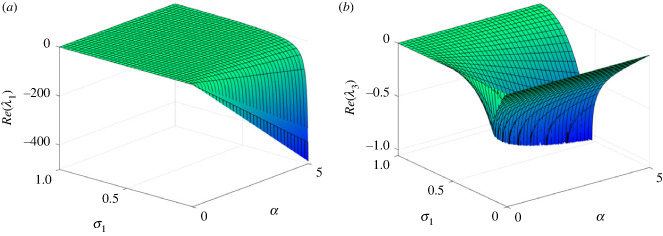
Plots of real parts of *λ*_1_ (*a*) and *λ*_3_ (*b*) as functions of *σ*_1_ and *α*. The functions are given in (B 1).

**Figure 12.**
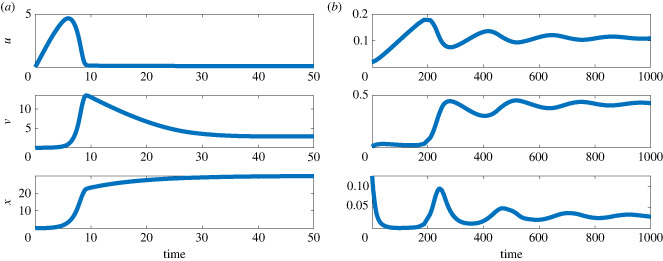
Simulations of the fast system (3.4). Panel (*a*) shows the dynamics of the stable node with *α* = 1 and *y*_0_ = 4. Panel (*b*) shows the dynamics of the stable spiral with *α* = 0.001 and *y* = 0.01. In both figures *ω* = 4.27, *σ*_1_ = 0.1 and *β* = 0.336. Further note that *βy*(0) − *α* > 0 is a biological restriction for the equilibrium.

## Data Availability

The Matlab codes are available from the Zenodo repository: https://doi.org/10.5281/zenodo.10685465 [46].

## Appendix A. Non-dimensionalization

To non-dimensionalize system (2.2), we begin by making the following substitutions of non-dimensional variables

A 1 $$\tau =\mu_{1}t,\;u=aS_{1},\;v=bS_{2},\;x=cX_{1},\;y=dX_{2},$$

and determine the appropriate values for *a*, *b*, *c* and *d* later to best separate timescales. The system becomes,

A 2 $$\begin{array}{rl} & \dot{u}=\frac{D}{\mu_{1}}(aS^{0}-u)-\frac{y_{1}a}{c}\frac{u}{aH_{1}+u}x,\\ & \dot{v}=-\frac{D}{\mu_{1}}v+\frac{y_{2}b}{c}\frac{u}{aH_{1}+u}x-\frac{y_{3}\mu_{2}b}{\mu_{1}d}\frac{v}{bH_{2}+v}y,\\ & \dot{x}=-\frac{1}{\mu_{1}}(D+k_{1})x+\frac{u}{aH_{1}+u}x\\ \;\mathrm{and}\; & \dot{y}=-\frac{1}{\mu_{1}}(D+k_{2})y+\frac{\mu_{2}}{\mu_{1}}\frac{v}{bH_{2}+v}y. \end{array}\}$$

Letting *a* = 1/*H*_1_, *b* = 1/*H*_2_ then yields

A 3 $$\begin{array}{rl} & \dot{u}=\frac{D}{\mu_{1}}(S^{0}/H_{1}-u)-\frac{y_{1}}{cH_{1}}\frac{u}{1+u}x,\\ & \dot{v}=-\frac{D}{\mu_{1}}v+\frac{y_{2}}{cH_{2}}\frac{u}{1+u}x-\frac{y_{3}\mu_{2}}{\mu_{1}dH_{2}}\frac{v}{1+v}y,\\ & \dot{x}=-\frac{1}{\mu_{1}}(D+k_{1})x+\frac{u}{1+u}x\\ \;\mathrm{and}\; & \dot{y}=-\frac{1}{\mu_{1}}(D+k_{2})y+\frac{\mu_{2}}{\mu_{1}}\frac{v}{1+v}y. \end{array}\}$$

Finally, taking *c* = *y*_2_/*H*_2_, *d* = *y*_3_*μ*_2_/*μ*_1_*H*_2_, $\epsilon =D/\mu_{1}$, $\alpha =\epsilon S^{0}/H_{1}$, *β* = *y*_1_/*cH*_1_, *σ*_1_ = *k*_1_/*μ*_1_, *σ*_2_ = *k*_2_/*D*, *ω* = *μ*_2_/*D*, we arrive at the simplified non-dimensional system

A 4 $$\begin{array}{rl} & \dot{u}=\alpha -\epsilon u-\beta \frac{u}{1+u}x,\\ & \dot{v}=-\epsilon v+\frac{u}{1+u}x-\frac{v}{1+v}y,\\ & \dot{x}=-\epsilon x-\sigma_{1}x+\frac{u}{1+u}x\;\\ \;\mathrm{and}\; & \dot{y}=-\epsilon (1+\sigma_{2})y+\epsilon \omega \frac{v}{1+v}y. \end{array}\}$$

## Appendix B. Stability of fast system

System (3.4) and that the eigenvalues of the linearized system around (*u**, *v**, *x**) are given by

B 1 $$\lambda_{1,2,3}=\left(\begin{array}{c} \frac{\sqrt{\alpha }\;(\sigma_{1}-1)\;\left(\sqrt{\alpha -2\;\alpha \;\sigma_{1}+\alpha \;{\sigma_{1}}^{2}-4\;{\sigma_{1}}^{2}}-\sqrt{\alpha }\;\sigma_{1}+\sqrt{\alpha }\right)}{2\;\sigma_{1}}\\ -\frac{{\left(\alpha -\beta \;y\right)}^{2}}{\beta^{2}\;y}\\ -\frac{\sqrt{\alpha }\;(\sigma_{1}-1)\;\left(\sqrt{\alpha -2\;\alpha \;\sigma_{1}+\alpha \;{\sigma_{1}}^{2}-4\;{\sigma_{1}}^{2}}+\sqrt{\alpha }\;\sigma_{1}-\sqrt{\alpha }\right)}{2\;\sigma_{1}} \end{array}\right).$$

Clearly, *λ*_2_ < 0, while the real parts of *λ*_1_ and *λ*_3_ are plotted in figure 11 to show that the real parts of each eigenvalue are negative for our chosen parameter range. Note that the plot of *Re*(*λ*_3_) shows an obvious cusp. When the curve describing this cusp is projected into the *α*–*σ*_1_ plane it represents the curve in which *λ*_3_ goes from being purely real to having non-zero imaginary parts. Thus, this cusp corresponds to a transition between stable node and a stable spiral. Furthermore, some samples of the fast dynamics are shown in figure 12 indicating this difference in dynamical behaviour (i.e. oscillatory, versus non-oscillatory) that corresponds to the two different qualitative aspects of the equilibrium.

## Appendix C. Parameters

**C.1. Generating synthetic data**

To create the synthetic data, we simulate model (4.2) with parameter values given in table 3.

**C.2. Transient end times in the synthetic data**

From the analysis of deterministic model, we have a good understanding that the transient dynamics end when the solution approaches the equilibrium point. Thus, in §4.2, we claimed that, using our *a priori* knowledge that the transient dynamics end when $|S_{1}(t_{i})-S_{1}^{*}|<\delta_{eq}$. For each time series, we assume a noise level, *σ*, when producing our synthetic time series. In this, we chose *δ*_*eq*_ = *σ* + 2. The values for *σ* range from 0.0005 to 5, and are given on the *y*-axis in each panel of figures 7–9.

## Appendix D. Predicted transient end times

For both the monitoring and control data case, we compute *R*^2^, as shown in figures 7–9 , as follows:

D 1 $$R^{2}=1-\sum_{k=1}^{n}\frac{{\left(t_{k,\mathrm{end}}-{\tilde{t}}_{k,\mathrm{end}}\right)}^{2}}{{\left({\tilde{t}}_{k,\mathrm{end}}-{\bar{\tilde{t}}}_{\mathrm{end}}\right)}^{2}},$$

where *t*_*k*,end_ is the computed transient end time, as in §4.2, ${\tilde{t}}_{k,\mathrm{end}}$ is the predicted transient end time using our method outlined in §5.2, and ${\bar{\tilde{t}}}_{\mathrm{end}}$ is the mean of ${\tilde{t}}_{k,\mathrm{end}}$.

Figures 13 and 14 show the heat map of Pearson’s correlation coefficient for the various levels of noise, error tolerance and sparsity of data using absolute and relative error, respectively. Additionally, sample correlation scatter plots are given in figures 15 and 16 to complement figures 7 and 8, respectively. The scatter plots correspond to the noise level and error tolerance that give the highest *R*^2^ value from each panel under the condition that the method was able to predict a transient end time for more than half of the generated time series. Additionally, since some generated error curves remain either above or below our error thresholds, *δ*_*a*_ and *δ*_*r*_, respectively, we show in figures 17 and 18 the proportional of time series the method was able to produce a prediction to complement the results regarding *R*^2^ and Pearson’s correlation coefficient.
